# Incidence, costs and outcomes of avoidable hospitalizations in a southern European country: is there room for improvement?

**DOI:** 10.1186/1753-6561-6-S4-P31

**Published:** 2012-07-09

**Authors:** R Soares-dos-Reis, JA Freitas, A Costa-Pereira

**Affiliations:** 1 Department of Health Information and Decision Sciences, Faculty of Medicine of the University of Porto, Portugal & CINTESIS, Centre for Research in Health Information Technologies, Faculty of Medicine of the University of Porto, Portugal

## Introduction

In healthcare, as in other areas, performance evaluation and outcomes monitoring are an ever increasing priority. This can be accomplished using quality indicators: monitoring and benchmarking tools which allow spatial and temporal comparisons. In the 1990's, following the work of John Billings and colleagues, the Agency for Healthcare Quality and Research (AHRQ), developed a set of area-level Prevention Quality Indicators (PQI), which can be calculated using readily available administrative data. PQIs are based on the concept of avoidable hospitalizations (AH): conditions for which adequate and timely primary care (PC) could have prevented or reduced the need for hospitalization (e.g. heart failure, hypertension, diabetes). We aim to apply these indicators to the Portuguese national health system (NHS), a state-financed universal coverage system, and ascertain time and regional trends and the AH financial and mortality burden.

## Methods

Indicators were extracted from a nationwide inpatient database comprising 8 million records (2000-2007) using an SPSS syntax based on AHRQ PQI's technical specifications. The country was divided into 5 regions, corresponding to the Health Administrative regions (ARS). Financial costs were calculated based on Diagnosis-Related Group price tables and cost-quality ratio was based on ARS expenses. In-hospital mortality and length-of-stay (LOS) was also computed. Rates were age and sex standardized using direct standardization.

## Results

The national AH total is 838.18/100,000 person-years, accounting for 9.7% of all hospitalizations. Elderly males were at highest risk for an AH. The best performing regions were Alentejo and the North, and the worst was the Centre. The North had the best quality-cost ratio. No clear time trends were observed. The mean AH mortality was 10% (database average 5%) and the average LOS was 2.4 days longer than the database average. The yearly direct cost of AH amounted to 200 million €.

## Conclusions

We conclude that Portuguese administrative data is suitable for PQI calculation. AH represent a significant proportion of total hospitalizations and carry a substantial direct financial burden. No direct relationship between ARS expenditure and PC quality, as evaluated by PQIs, was found. Given the important regional asymmetries, there is certainly room for improving PC quality and abrogating these differences.

